# miR-552: an important post-transcriptional regulator that affects human cancer

**DOI:** 10.7150/jca.46613

**Published:** 2020-08-27

**Authors:** Yuhao Zou, Xin Zhao, Yin Li, Shiwei Duan

**Affiliations:** Medical Genetics Center, Ningbo University School of Medicine, Ningbo, Zhejiang, China.

**Keywords:** miR-552, cancer, diagnosis, prognosis, drug resistance

## Abstract

MiR-552 is a small non-coding RNA located on chromosome 1p34.3, and its expression level is significantly up-regulated in tissues or cells of various tumors. miR-552 can target multiple genes. These targeted genes play important regulatory roles in biological processes such as gene transcription and translation, cell cycle progression, cell proliferation, apoptosis, cell migration, and invasion. Besides, miR-552 may affect the efficacy of various anticancer drugs by targeting genes such as TP53 and RUNX3. This review summarizes the biological functions and clinical expressions of miR-552 in human cancer. Our goal is to explore the potential value of miR-552 in the diagnosis, prognosis, and treatment of human cancer.

## Introduction

MicroRNAs (miRNAs) are a class of non-coding small RNAs with a length of about 22 bp. They mostly bind to the 3 'untranslated region (3'UTR) of the mRNA, thereby regulating the expression of most genes after transcription 1. Compared with normal tissues, miR-552 expression is up-regulated in many tumors, including colorectal cancer (CRC) 2, Hepatocellular carcinoma (HCC) 3, ampulla adenocarcinoma 4, gastric cancer (GC) 5, and rectal adenocarcinoma (READ) 5, osteosarcoma (OS) 6, lung cancer (LCA) 7, papillary mucinous tumor (IPMN) 8, and ovarian cancer (OC) 9. By targeting multiple genes, miR-552 can promote cell cycle progression 10, cell proliferation, migration, and invasion 3, thereby leading to tumorigenesis and cancer development. At the same time, miR-552 can also play a role in suppressing tumors by promoting apoptosis 11. miR-552 can affect the resistance of cancer cells to anti-cancer drugs by targeting SMAD2, TP53, and RUNX3. Here, this review summarizes miR-552-related biological functions and mechanisms in human tumors. We aim to improve our understanding of its clinical significance in the diagnosis, prognosis, and treatment of tumors.

## miR-552 family

**Figure 1:**
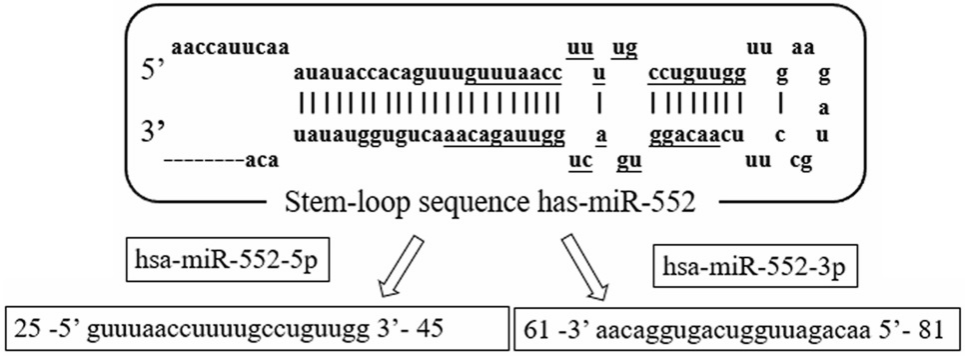
** The sequence structure of miR-552 family.** Hsa-miR-552 is located on chromosome 1(chr1: 189966305-189966399). It has two mature sequences, hsa-miR-552-5p (miR-552) and hsa-miR-552-3p.

The miR-552 family is located on chromosome 1 (chr1: 189966305-189966399). The family includes two main members of the human genome, which are defined by hsa-miR-552-5p (miR-552) and hsa-miR-552-3p, respectively. Both mature sequences are 21 nucleotides in length and are highly conserved (**Figure 1**). Currently, most hsa-miR-552 studies are related to miR-552. Based on the information of miRbase, the sequence of miR-552-5p (miR-552) is guuuaaccuuuugccuguugg, and the sequence of miR-552-3p is aacaggugacugguuagacaa.

## The molecular function of miR-552 target gene

MiR-552 regulates the expression of various genes after transcription by directly binding to the 3′UTR of the target gene mRNA. The target genes of miR-552 have unique molecular functions, such as catalytic activity, transcriptional regulation, and binding.

Adhesion junction-associated protein 1 (AJAP1) is described as a novel component of adhesion junctions in polarized epithelial cells. Some studies have suggested that AJAP1 may bind to each other through β-catenin and E-cadherin-mediated binding complexes 12. Wnt inhibitor factor 1 (WIF-1) is a tumor suppressor gene that mainly encodes secreted proteins. Normally, it can inhibit the Wnt /β-catenin pathway by binding to Wnt protein signal transduction 13. TIMP2 is a natural inhibitor of matrix metalloproteinases. Matrix metalloproteinases are a group of peptidases involved in extracellular matrix degradation. TIMP2 can preferentially bind to proMMP-2 14. RUNX3 is a member of the RUNX family of transcription factors. It can inhibit the transcription of superoxide dismutase 3 (SOD3) by binding to the SOD3 promoter to induce the production of reactive oxygen species, thereby affecting the apoptosis of CRC 15.

## The clinical significance of miR-552 in human cancer

Current research shows that miR-552 is up-regulated in at least 12 cancers (**Table 1**). Compared with adjacent tissues, the expression level of miRNA-552 in colorectal cancer (CRC) tissues was up-regulated 16. This is consistent with the results of five previous independent studies 2, 17-19. And the miR-552 is expressed in CRC more than three times the normal tissue 16, 20-23. The miR-552 expression was also significantly up-regulated in various CRC cell lines including HCT116, HT-29, HCT-15, and HT-29 21-24. Consistent with this, miR-552 was also found to be up-regulated in the serum of CRC patients 25. Comparing miR-552 expression in 158 pairs of colon cancer (CC) adenocarcinoma tissues with proficient DNA mismatch repair (pMMR) and adjacent tissues found that miR-552 expression was significantly associated with CC risk 20. In liver cancer (HCC), Qu W et al. detected the expression levels of miR-552 in 81 pairs of HCC tissues and adjacent non-tumor tissues and found that miR-552 expression levels in HCC tissues were increased 3, which was found in the other two independent studies 26, 27. Han T et al. found that miR-552 was overexpressed in liver tumor-initiating cells (T-ICs) 28. In 107 cases of ampullary adenocarcinoma, the expression of miRNA-552 was also significantly up-regulated 4. Through the comparison of 122 gastric cancer (GC) tissues and matched adjacent tissues, Feng X et al found that miR-552 was up-regulated in GC tissues. This result was found in various GC cell lines (AGS, MGC-803, and MKN-45) 29. In rectal adenocarcinoma (READ), Lai CH et al. found that the expression of miR-552 in READ tissues (n=162) was much higher than normal tissues (n=3) in TCGA database 30. By comparing the expression of miR-552 between 51 osteosarcomas (OS) tissues and 19 adjacent normal tissues, it was found that the expression level of miR-552 was significantly increased in OS tissues 6. This was confirmed by other OS studies 31, 32. Kim HK et al. found that miR-552 was up-regulated in 35 lung cancer (LCA) tissues 7. And miR-552 expression was significantly increased in two paraffin-embedded intraductal papillary mucinous tumor (IPMN) tissues and four pancreatic cancer cell lines (Panc1, MiaPaCa-2, XPA-3, BxPC-3, and HPNE) 8. In ovarian cancer (OC), Zhao W et al. found that the expression of miR-552 in OC tissues and cells (HO8910 and HGSOCO) was upregulated 9 compared with non-tumor tissues and non-tumor cell lines. In pancreatic cancer (PC), miR-552 is up-regulated in PC tissues and cells (CFPAC-1, AsPC-1, MIA-PaCa2, Capan-2, BXPC-3, and PANC-1) compared to normal PC and cell lines 33. Gu J et al. found that miR-552 was up-regulated in 20 cases of laryngeal carcinoma and four laryngeal cancer cell lines (M2E, M4E, TU212, and Hep-2). In addition, they also found that miR-552 expression level was higher in stage 3/4 laryngeal cancer patients than that in patients with stage 1/2 laryngeal carcinoma 34. Through microarray analysis of 29 oral squamous cell carcinoma (OSCC) samples, Mayakannan Manikandan et al. found that miR-552 was up-regulated in OSCC 35.

Compared with normal tissues, the fold increase of miR-552 expression is different in different cancer tissues. MiR-552 is expressed in CRC more than three times the normal tissue 16, 20-23. The expression of miR-552 in HCC is more than twice that of normal tissues 3. The expression of miR-552 in READ is 3.20 times that of normal tissues 30. Compared with normal tissues, the expression level of miR-552 in metastatic lung adenocarcinomas is 39 times higher 7. The expression of miR-552 in IPMN is more than three times that of normal tissues 8. Also, the expression of miR-552 in OS cells was 5.01 times that of normal cells 32.

These results indicate that the abnormal expression of miR-552 in cancer tissues and cells may be closely related to the occurrence and development of cancer.

## The biological role of miR-552 in human cancer

### Regulation of gene transcription and translation

As a member of the CYP family, CYPE1 can convert carcinogens and inactive xenobiotics into reactive metabolites 36. Studies have found that the cruciform structure in gene promoters is usually involved in transcriptional regulation 37. miR-552 can form DNA-RNA hybrids with CYP2E1 and regulate the expression of CYP2E1, thereby inhibiting the transcription and translation in the HCC cell nucleus and cytoplasm 38. In HCC HepG2 cells, the non-seed region of nuclear miR-552 (AACAGAUUGGUCA) binds to the cruciform structure of the CYP2E1 promoter and inhibits transcription of CYP2E1, while the seed region of cytoplasmic miR-552 (GUGGACAA) can bind the 3' UTR of CYP2E1 mRNA and thus inhibit translation of CYP2E1 38.

### Cell cycle regulation

The cell cycle is an important process of cell proliferation. The abnormal cell cycle is closely related to the proliferation of cancer cells.

MiR-552 inhibits the expression of WIF1 and may promote the cell cycle of glioblastoma cells 10. Down-regulation of WIF1 expression induced by miR-552 can cause glioblastoma to significantly reduce the number of cells in the S phase and increase the number of cells in the G1 phase, thereby inhibiting cell growth and differentiation 10. Cyclin D3 (CCND3) is part of the D-type cyclin and mainly regulates the G1/S phase transition of the cell cycle 39, 40. In CRC, miR-552 inhibitors can decrease the expression of cyclin and c-Myc 19.

### Cell proliferation and apoptosis

**Figure 2:**
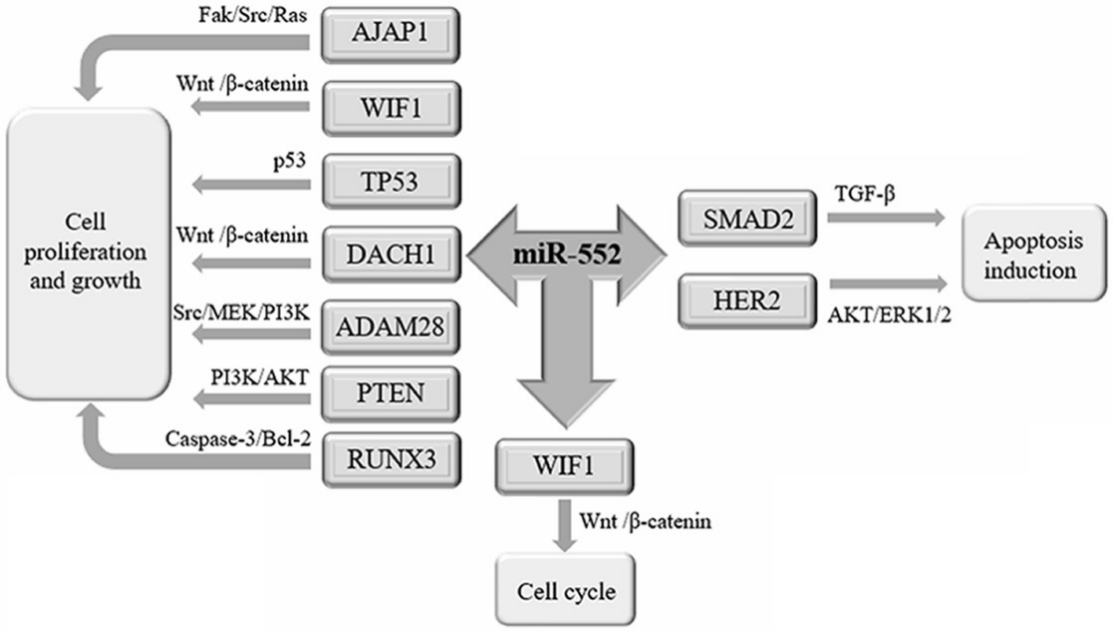
** The biological function of miR-552 in cancer.** miR-552 is involved in the development and progression of tumors through its target genes. miR-552 targets AJAP1, WIF1, TP53, DACH1, ADAM28, PTEN, and RUNX3 to promote cell proliferation and growth. miR-552 targets SMAD2, HER2 promotes apoptosis. By inhibiting the expression of WIF1, miR-552 can promote the cell cycle of cancer cells.

Recent studies have shown that miR-552 is related to the proliferation of cancer cells (**Figure 2**).

The Src pathway is an important signaling pathway that mediates the proliferation of cancer cells 41, while the AJAP1is inversely related to the expression of Src protein 42. Overexpression of miR-522 can inhibit AJAP1, thereby increasing the level of Src protein suppressed by AJAP1, leading to a large number of HCC cell proliferation 3. Abnormal Wnt/β-catenin pathway may lead to cell proliferation and malignant transformation 43, 44, of which WIF1 plays a tumor-suppressive role by directly binding to Wnt protein 45. In HCC, miR-552 binds to the 3'-UTR of WIF1 mRNA to form an RNA-induced silencing complex (RISC), which inactivates the Wnt/β-catenin signaling pathway to promote cell proliferation 27. HER2 belongs to the receptor tyrosine kinase of the epidermal growth factor receptor (EGFR) family 46. In contrast, miR-552 can inhibit HER2 expression by directly binding 3'-UTR of HER2 47, and induce breast cancer cell apoptosis 11.

As a transcription factor, p53 plays a central role in tumor suppression mainly through the transcriptional regulation of many target genes 48. miR-552 can down-regulate the expression of p53 and promote cell proliferation, thereby showing oncogenic properties in the CRC and GC 24, 34.

DACH1 is a tumor suppressor gene that has been shown to be down-regulated in a variety of diseases such as breast cancer, prostate cancer and endometrial cancer 49. In CRC, miR-552 inhibits DACH1 expression at the post-transcriptional level and enhances the function of the Wnt/β-catenin signaling pathway to promote CRC cell proliferation 19.

ADAM28 is a transmembrane and secreted protein of the A integrin and metalloproteinase (ADAM) family, and it can affect cell adhesion and migration 50. ADAM28 is involved in the growth and metastasis of solid tumors and the progress of hematological malignancies 51. miR-552 can directly target ADAM28 and inhibit its expression, thereby promoting CRC cell proliferation through the Src/MEK/PI3K signaling pathway 18.

As a highly homologous protein, SMAD2 is a mediator that mediates multiple signaling pathways and is a direct mediator of transforming growth factor (TGF-β) 52. Activation of the TGF-β signaling pathway provides growth-suppressing signals in the normal intestinal epithelium 53. When miR-552 targets the inhibition of SMAD2, the growth of 5-FU resistant CRC tumor cells can be inhibited by TGF-β 2. PTEN is a dual-function phosphatase and tensin homolog located on chromosome 10 and is a tumor suppressor gene associated with a variety of malignancies 54. A study has shown that miR-552 down-regulates PTEN and activates AKT phosphorylation in liver T-ICs, which is conducive to promoting liver T-ICs expansion 28. Zhao W et al found that miR-552 can directly regulate PTEN expression through its 3'-UTR interaction and promote the development of OC cells 9.

Bcl-2 can inhibit the dimerization of Bax, thereby reducing caspase-3-mediated apoptosis 55, 56. RUNX3 is a tumor suppressor gene that regulates gene expression related to cell viability 57. Studies have found that miR-552 can increase the expression of Bcl-2 in HCC cells by targeting RUNX3, and reduce the expression of caspase3 and Bax, thereby inhibiting the apoptosis of HCC cells 26.

### EMT

**Figure 3:**
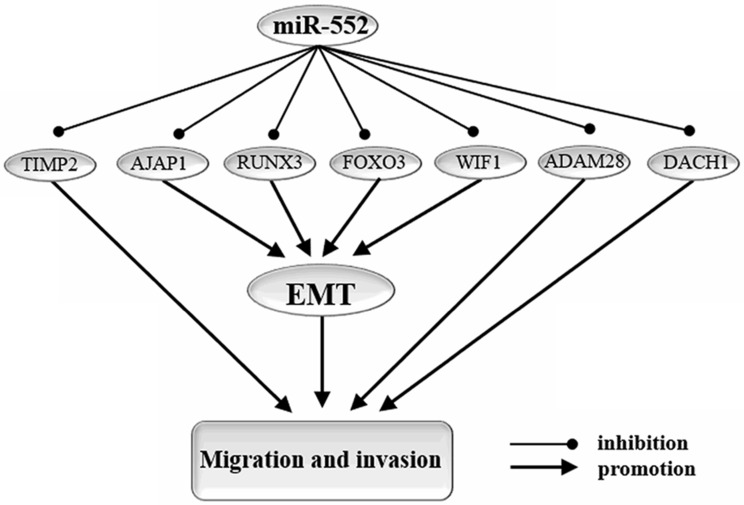
** miR-552 targets genes that affect cell migration, invasion, and EMT.** EMT: epithelial-to-mesenchymal transition. By targeting AJAP1, RUNX3, WIF1, FOXO3, miR-552 reverses the process of EMT and thereby inhibits migration and invasion of cancer cells. In addition, miR-552 regulates cell migration and invasion in cancer through other pathways. miR-552 promotes cell migration and invasion in cancer by targeting TIMP2, ADAM28, and DACH16.

Epithelial-mesenchymal transition (EMT) is an important biological process for epithelial cells-derived malignant tumor cells to acquire the ability to migrate and invade 58 (**Figure 3**).

MiR-552 can promote the development of EMT by targeting AJAP1 3, WIF1 27, RUNX3 26, and FOXO3 33. In HCC cells, miR-552 increased the expression level of E-cadherin and down-regulated the expression of N-cadherin and vimentin by down-regulating the expression of AJAP1 3. In the HCC HIF3B cell line, miR-552 could down-regulate WIF1 expression, increase the effect of E-cadherin, and promote the EMT pathway 27. miR-552 can target RUNX3 by inhibiting miR-186/E-cadherin/EMT pathway, thereby promoting cell migration and invasion of HCC cells 26, 57. Forkhead box protein O3 (FOXO3) is a member of the Forkhead box-O transcription factor, which participates in the EMT process by regulating the Wnt signaling pathway and has been shown to play a key role in PC 59, 60. miR-552 can up-regulate the expression of FOXO3 in PC and inhibit the Wnt signaling pathway, thereby inhibiting EMT and PC cell migration 33.

### Cell migration and invasion

Cell migration and invasion are important features of various cancers and are the main causes of high cancer mortality 26. miR-552 can promote the expression of MMP, which significantly promotes the migration and invasion of OS MG63 cells 31. As a member of the TIMP family, TIMP2 can inhibit MMP, thereby reducing the degradation of the extracellular matrix and inhibiting the migration and invasion of primary tumor cells 61, 62. ADAM28 acts as a "signaling scissor" in various membrane environments 50, 63. miR-552 targets the ADAM28 gene to promote the migration and invasion of CRC cells, thereby enhancing the carcinogenesis effect 18. Increased expression of DACH1 can inhibit the abundance of c-Myc 19. miR-552 activates the Wnt/β-catenin signaling pathway by regulating DACH1 expression, thereby promoting the proliferation and migration of CRC 19.

### miR-552 and drug resistance in cancer cells

The antimetabolite drug 5-fluorouracil (5-FU) can improve the 12-month survival rate of CRC patients 64. SMAD2 phosphorylation can promote the occurrence of cancer and increase cell resistance 65. The miR-552/SMAD2 cascade plays a key role in regulating the response of cells to 5-FU chemotherapy. miR-552 can down-regulate the expression of SMAD2, and the low expression of miR-552 and the high expression of SMAD2 can play a synergistic role to promote 5-FU resistance of cancer cells, which is not conducive to the treatment of CRC 2. However, miR-552 is significantly up-regulated in CRC side population (SP) cells which are resistant to multiple cancer drugs 22.

TP53 is an important tumor suppressor. The wild-type TP53 neuroblastoma cell line is very sensitive to the synergistic treatment of doxorubicin and GSK2830371, an antagonist of phosphatase 1 (WIP1) 66. Targeted inhibition of TP53 by miR-552 may reduce the sensitivity of neuroblastoma cell lines to drugs, thereby inhibiting the effect of drugs on cancer cells 24.

Increased methylation of the cancer suppressor gene RUNX3 reduces RUNX3 gene expression, which increases the risk of CRC 67. In CRC, methylation of RUNX3 can inhibit the therapeutic effect of the chemotherapeutic drug irinotecan, resulting in a poor prognosis for patients with CRC 68. Targeted inhibition of RUNX3 by miR-552 may inhibit the therapeutic effect of chemotherapy drugs 26.

Celecoxib is an inhibitor of cyclooxygenase-2 (COX-2), which can inhibit the growth of tumor cells 69, 70. When celecoxib was used to treat HT-29 CRC cells, the expression of miR-552 was down-regulated by 2.1 times compared with control cell lines, which was beneficial to the treatment of CRC 71.

## miR-552-3p and human cancer

MiR-552-3p is the antisense strand of miR-552, and there is little research on miR-552-3p and cancer. Wei Z et al. analyzed miRNA-Seq data of GC and found that compared with normal tissues, miR-552-3p expression was up-regulated nearly 3.55 times in GC 72. Survivin is a well-known target for cancer treatment. Fengzhi Li et al. found from the GeneGo database that miR-552-3p can be combined with survivin transcription to inhibit cancer progress, which is helpful for anti-cancer against survivin 73. And some studies have found that circFUT8 may competitively bind to miR-552-3p, thereby eliminating the inhibition of related target genes and related to the progress of HCC 74. Overexpression of miR-552-3p can reduce the level of CCND3, thereby inhibiting the cell cycle and proliferation of NCI-H460 LCA cells 75. However, the specific mechanism of miR-552-3p remains to be studied.

## miR-552 and cancer prognosis

MiR-552 is significantly associated with cancer prognosis in many studies. The expression of miR-552 was up-regulated in CRC tumor tissues, and the up-regulation of miR-552 expression was significantly associated with decreased survival in CRC patients 16. miR-552 is significantly upregulated in CC resistant cells (SP cells) 22. Besides, in CRC patients receiving 5-FU chemotherapy, the down-regulated miR-552 expression can increase overall survival in CRC patients 2. In HCC cells, miR-552 overexpression can target the inhibition of AJAP1, which promotes the migration, invasion, and EMT of HCC cells, and is associated with poor prognosis in HCC patients 3. The high expression of miR-552 in HCC is associated with malignant clinicopathological features and decreased survival 27. High levels of miR-552 are associated with poor disease-free survival (DFS) and overall survival in patients 28. The upregulation of miR-552 is also associated with advanced TNM stage and lymph node metastasis of GC 5. miR-552 is an independent prognostic factor in patients with GC, and its upregulation is significantly associated with tumors with advanced TNM staging, lymph node metastasis, intestinal metaplasia, and genomically stable subtypes of tumors. Moreover, in GC patients with high expression of miR-552, the overall survival time is lower than that of patients with low expression 29. The miR-552 expression is up-regulated in OS tissues and cell lines, which inhibits TIMP2 to promote cell migration and invasion, and ultimately leads to poorer prognosis for patients with OS 31. Xiaobo Shi et al. used ten characteristic RNAs including miR-552 for esophageal squamous cell carcinoma (ESCC) prognostic scoring and found that miR-552 is a dangerous RNA that causes ESCC 76.

## Regulation of miR-552 transcription

NGX6 is a tumor suppressor gene that plays an important role in nasopharyngeal and CRC 77, 78. NGX6 is able to inhibit Cyclin D1 expression, thereby delaying the cell cycle and promoting apoptosis 77. Wang XY et al. found that NGX6 could up-regulate the expression of miR-552 21. NGX6 was found to participate in the regulation of tumor cell apoptosis, migration, and other functions through the Notch/JNK molecular signaling pathway 21. miR-552 is considered to be an important target for Linc00261 in PC. Chen T et al. found that Linc00261 can upregulate the expression of FOXO3 by inhibiting the expression of miR-552 to inhibit the Wnt signaling pathway, and finally weaken the EMT and cell migration process of PC 33.

## Conclusions

This review summarizes the research progress of miR-552 in human cancer. The target genes of miR-552 have a series of molecular functions, such as catalytic activity, transcriptional regulation, and binding. The expression of miR-552 is up-regulated in at least 12 cancers, which makes it possible to become a molecular marker for cancer diagnosis. In addition, miR-552 can also be used as a prognostic marker in cancer patients, because the increased expression of miR-552 in cancer is related to the poor survival level of patients. In cancer, miR-552's target genes are involved in a variety of biological processes, such as transcription and translation, cell cycle, cell proliferation and apoptosis, EMT, cell migration and invasion. miR-552 can also suppress or promote the effects of cancer drugs by targeting genes. In summary, miR-552 plays a significant role in the initiation and progression of key biological and pathological processes in most of the human cancer. miR-552 can also be used for the diagnosis and prognosis of various cancers. In addition, miR-552-3p can also inhibit the cell cycle and cell proliferation of lung cancer cells. However, the current research on miR-552-3p is still lacking. In the future, it is necessary to explore its relationship with more cancers.

## Figures and Tables

**Table 1:** miR-552 is up-regulated in multiple cancer tissues and cells

Cancer type	Tissues or cell lines	Target gene	Reference
CRC	183 CRC tissues and 183 matched normal tissues		61
	20 CRC tissues and 183 matched normal tissues	DACH1	19
	50 CRC tissues and 50 matched normal tissues	ADAM28	18
	97 CRC tissues	SMAD2	2
	Cell (HCT116)	TP53	24
	Cell (HT-29)		21
	6 CRCs and 6 matched normal tissues; Cell (HT-29)		23
	Cells (HCT-15, HT-29)		22
	55 normal tissues, 11 IBD-dysplasias, and 38 frank IBD-cancers		17
	44 mCRC blood samples		25
	158 pMMR adenocarcinomas and 64 adenocarcinomas dMMR		20
HCC	81 pairs of HCC tissues and matched adjacent tissues	AJAP1	3
	76 pairs of HCC tissues and matched adjacent tissues	WIF1	27
	15 pairs of HCC and matched adjacent tissues; Cell (HCC)	RUNX3	26
	110 HCC tissues; Cell(T-Ics)	PTEN	28
Ampullary Adenocarcinoma	107 ampullary adenocarcinomas		4
GC	122 pairs of GC tissues and matched adjacent tissues; Cells (GES-1 and AGS, MGC-803, MKN-45)		5
READ	162 READ tissues and 3 normal tissues		30
OS	51 OS tissues and 19 adjacent normal tissues	WIF1	6
	67 OS tissues and 67 adjacent normal tissues	TIMP2	31
	323 OS Cells		32
LCA	35 lung carcinomas tissues and 2 adjacent normal lung tissues		7
IPMN	2 IPMN tissues; Cells (Panc1, MiaPaCa-2, XPA-3, BxPC-3, HPNE)		8
OC	80 pairs of OC tissues and matched adjacent normal tissues; Cells (HO8910, HGSOCO)	PTEN	9
PC	54 PC tissues and 54 adjacent normal tissues; Cells (CFPAC-1, AsPC-1, MIA-PaCa2, Capan-2, BXPC-3, PANC-1)	FOXO3	33
Laryngeal carcinoma	20 laryngeal carcinoma tissues; Cells (M2E, M4E, TU212 and Hep-2)		34
OSCC	29 OSCC tissues and 61 verification sample		35

CRC: colorectal cancer; HCC: hepatocellular carcinoma; GC: gastric cancer; READ: rectal adenocarcinoma; OS: osteosarcoma; LCA: lung cancer; IPMN: intraductal papillary mucinous neoplasms; OC: ovarian cancer; PC: pancreatic cancer; OSCC: oral squamous cell carcinoma.
